# Are We Estimating the Mean and Variance Correctly in the Presence of Observations Outside of Measurable Range?

**DOI:** 10.1002/prp2.70048

**Published:** 2024-12-20

**Authors:** Markéta Janošová, Stanislav Katina, Jozef Hanes

**Affiliations:** ^1^ Department of Mathematics and Statistics Masaryk University Faculty of Science Brno Czech Republic; ^2^ Slovak Academy of Sciences Institute of Neuroimmunology Bratislava Slovakia

**Keywords:** clinical trial, estimation techniques, limit of detection, limit of quantitation, measurable range, simulation study

## Abstract

Laboratory measurements used for safety assessments in clinical trials are subject to the limits of the used laboratory equipment. These limits determine the range of values which the equipment can accurately measure. When observations fall outside the measurable range, this creates a problem in estimating parameters of the normal distribution. It may be tempting to use methods of estimation that are easy to implement, however selecting an incorrect method may lead to biased estimates (under‐ or overestimation) and change the research outcomes, for example, incorrect result of two‐sample test about means when comparing two populations or biased estimation of regression line. In this article, we consider the use of four methods: ignoring unmeasured observations, replacing unmeasured observations with a multiple of the limit, using a truncated normal distribution, and using a normal distribution with censored observations. To compare these methods we designed a simulation study and measured their accuracy in several different situations using relative error $\frac{\hat{\mu }-\mu }{\mu }$, ratio $\frac{\hat{\sigma }}{\sigma }$, and mean square errors of both parameters. Based on the results of this simulation study, if the amount of observations outside of measurable range is below 40%, we recommend using a normal distribution with censored observations in practice. These recommendations should be incorporated into guidelines for good statistical practice. If the amount of observations outside of measurable range exceeds 40%, we advise not to use the data for any statistical analysis. To illustrate how the choice of method can affect the estimates, we applied the methods to real‐life laboratory data.

## Graphical abstract

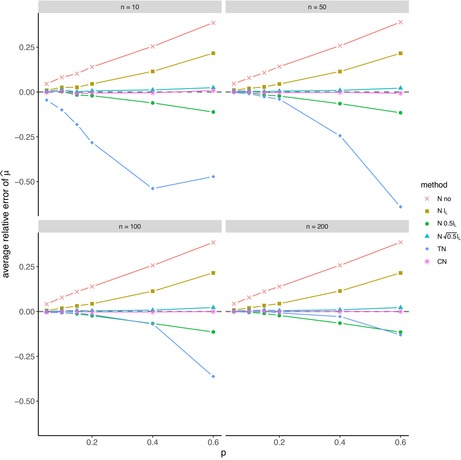

## 1 Introduction

During clinical trials, various laboratory data need to be measured and assessed. This includes routine safety laboratory data (e.g., erythrocytes, leukocytes, troponin T, creatinine, bilirubin), pharmacokinetic data, which relate to the concentration of a drug after its administration, or biomarkers, which include indicators of a disease or its severity and indicators of probable treatment efficacy (e.g., proteomic data, inflammatory markers such as C‐reactive protein [CRP], immunoglobulins). These data are measured repeatedly on pre‐specified visits, and it is important to assess them correctly. To ensure reliable and reproducible data, laboratories need to follow Good Laboratory Practice [1, 2]. This includes maintenance and regular calibration of laboratory equipment and validation of analytical methods. However, even properly calibrated laboratory equipment has limits to what it can accurately measure. Consequently, researchers must be prepared that during the statistical analysis, they might encounter data with observations below or above measurement limits.

Regulatory bodies, such as European Medicines Agency (EMA) or U.S. Food and Drug Administration (FDA), require a statistical analysis plan (SAP) to be prepared at the beginning of the clinical trials. This plan should describe not only the trial design and intended methods for statistical analysis but also how missing data would be dealt with. Removing observations with missing data from the analysis is not an acceptable approach. Using an incorrect method of handling missing data may lead to biased estimates (under‐ or overestimation) and change the research outcomes.

Guidelines stipulate that removing observations with missing data from the analysis is not an acceptable approach. Because the reasons for missing data are varied, no specific methods are encouraged above others and it is recommended to conduct sensitivity analyses to evaluate the robustness of the methods. However, the guidelines focus mainly on missing data due to dropout or nonadherence to the study protocol [3, 4, 5, 6, 7].

Regarding observations subject to measurement limits, SAPs often contain statements like “laboratory values of the form of ‘< LLoQ’ or ‘> ULoQ’ will be imputed, respectively, as ‘LLoQ’ or ‘ULoQ’,” “pharmacokinetic values of the form of ‘< LLoQ’ or ‘> ULoQ’ will be imputed as ‘NA’” or “biomarker values of the form of ‘< LLoQ’ will be imputed as ‘0’,” where LLoQ and ULoQ are, respectively, the Lower and Upper Limit of Quantitation. All three statements reflect using methods that bias the estimates of mean and standard deviation and therefore the introduction of rules, how to handle data outside of measurable range, into guidelines for good statistical practice in clinical trials should be considered.

During clinical trials, we might encounter different limits of laboratory equipment. These include Limit of Blank (LoB), Limit of Detection (LoD), LLoQ, and ULoQ. The LoB is determined from a series of measurements of analyte‐free samples. Based on a false positive rate of 5%, LoB is set to the 95th percentile value of the analyte‐free samples, meaning that 95% of analyte‐free samples are correctly identified as such [8].

For samples with an amount of analyte equal to LoB, 50% would be identified as false negatives if decisions were made according to LoB. The LoD is based on LoB and the standard deviation of low‐concentration samples to ensure a false negative rate of 5%. LoD is the lowest amount of analyte which is correctly detected in 95% of samples [8, 9].

The LLoQ is the lowest amount of analyte that can not only be detected but also measured with a specified accuracy. The ULoQ is the highest amount of analyte that can be measured with a specified accuracy [8, 9, 10, 11].

If a biological sample has such an amount of analyte, that it falls below lower limit $l_{L}$ or above upper limit $l_{U}$, that is all the information we can obtain. Depending on the specific situation and available information, $l_{L}$ will be LoB, LoD, or LLoQ, and $l_{U}$ will be ULoQ. In some situations, it is possible to dilute a sample that exceeds $l_{U}$ and reanalyze it, but this may affect the accuracy [10, 11].

Assuming that the analyte follows a normal distribution with mean μ and standard deviation σ, making an inference about the whole population, not just those within the measurable range, presents us with a problem of estimating parameters of the distribution. Since the objective is to get parameter estimates to be able to conduct parametric inference about μ and σ, we do not consider nonparametric methods. Because the observations outside of measurable range are in their nature censored, and therefore they are missing not at random, multiple imputation methods are not suitable either, as they require the data to be missing completely at random or missing at random.

Several methods can be used to address the issue of estimating parameters μ and σ. The first approach (Method 1) is to completely disregard the truncated observations and estimate the parameters of normal distribution using only the remaining observations. This leads to a decrease in sample size and an underestimated standard deviation. For samples subjected only to a lower limit, the mean will be overestimated, and for samples subjected only to an upper limit, the mean will be underestimated. Another approach (Method 2) is to replace the truncated observations with the limit or its multiple (e.g., 0.5 or $\sqrt{0.5}$ of $l_{L}$, or 1.5 or $1+\sqrt{0.5}$ of $l_{U}$). In practice, some researchers replace values below $l_{L}$ by 0, but this choice leads to heavily underestimated mean and overestimated standard deviation (as an extreme case of Method 2 with 0 times $l_{L}$), and therefore we do not include this option in this study. The third approach (Method 3) is to use a truncated normal distribution $\mathrm{TN}\left(\mu ,\sigma^{2},l_{L},l_{U}\right)$ instead of a normal distribution. The last approach (Method 4), that we will consider, is to use a normal distribution with censored observations, where values below $l_{L}$ or above $l_{U}$ can be considered censored.

To determine which of these methods are suitable, we conducted a simulation study to examine the relationship of the estimate's accuracy to the amount of truncated observations for various sample sizes. Accuracy of estimates is measured through the relative error $\left(\hat{\mu }-\mu \right)/\mu$ for the estimate of the mean, through the ratio $\hat{\sigma }/\sigma$ for the estimate of the standard deviation and through mean square errors (MSE) for both of the parameters.

## 2 Methods

The sample size (total number of observations) is denoted as n, the number of truly measured observations is denoted as $n_{o}$ and the number of truncated observations (observations below $l_{L}$ or above $l_{U}$) is denoted as $n_{t}$. It follows that $n=n_{o}+n_{t}$ and that the proportion of truncated observations is $p=n_{t}/n$. Let us denote the truly measured observations as $x_{o,1},x_{o,2},\ldots ,x_{o,n_{o}}$, the truncated observations as $x_{t,1},x_{t,2},\ldots ,x_{t,n_{t}}$, and the vector of all observations as $x={\left(x_{o,1},x_{o,2},\ldots ,x_{o,n_{o}},x_{t,1},x_{t,2},\ldots ,x_{t,n_{t}}\right)}^{T}$.

### 2.1 Method 1: Ignoring Truncated Observations

This approach simply ignores truncated observations and only works with truly measured observations [12]. Maximum likelihood estimates of the parameters are then directly calculated as

$$\hat{\mu }=\frac{1}{n_{o}}\sum_{i=1}^{n_{o}}x_{o,i},\;\hat{\sigma }=\sqrt{\frac{1}{n_{o}}\sum_{i=1}^{n_{o}}{\left(x_{o,i}-\hat{\mu }\right)}^{2}}.$$

An obvious disadvantage is the decrease of sample size, especially with a high proportion of truncated observations, and the standard deviation σ will be underestimated. For samples subjected only to a lower limit, overestimation of μ can be expected, whereas for samples subjected only to an upper limit, underestimation of μ can be expected.

### 2.2 Method 2: Replacing Truncated Observations

This approach replaces truncated observations by a multiple of the limit: [12, 13, 14, 15, 16, 17].

$$x_{r,j}=\left\{\begin{array}{ll} c_{L}l_{L}, & \text{if}\;x_{t,j}<l_{L},\\ c_{U}l_{U}, & \text{if}\;x_{t,j}>l_{U}. \end{array}\right.$$

To replace with the limit, we set the constants $c_{L}=c_{U}=1$. Other commonly used options are $c_{L}=0.5$ and $c_{U}=1.5$, or $c_{L}=\sqrt{0.5}$ and $c_{U}=1+\sqrt{0.5}$. Maximum likelihood estimates of the parameters are then directly calculated as

$$\hat{\mu }=\frac{1}{n}\left(\sum_{i=1}^{n_{o}}x_{o,i}+\sum_{j=1}^{n_{t}}x_{r,j}\right),\hat{\sigma }=\sqrt{\frac{1}{n}\left(\sum_{i=1}^{n_{o}}{\left(x_{o,i}-\hat{\mu }\right)}^{2}+\sum_{j=1}^{n_{t}}{\left(x_{r,j}-\hat{\mu }\right)}^{2}\right)}.$$

While this method retains the original sample size, underestimation of σ can be expected when using the replacement by the limit. For samples subjected only to a lower limit, overestimation of μ can be expected if $c_{L}=1$, whereas for samples subjected only to an upper limit, underestimation of μ can be expected if $c_{U}=1$. If multiples $c_{L}=0.5$ and $c_{U}=1.5$, or $c_{L}=\sqrt{0.5}$ and $c_{U}=1+\sqrt{0.5}$, are used, such immediate statements cannot be made.

### 2.3 Method 3: Using a Truncated Normal Distribution Instead of a Normal Distribution

A truncated normal distribution takes into account the limits that the observations are subjected to [18, 19]. However, it does not account for the number of truncated observations. The density $f_{\mathrm{TN}}\left(x\right)$ of truncated normal distribution $\mathrm{TN}\left(\mu ,\sigma^{2},l_{L},l_{U}\right)$ is defined as

$$f_{\mathrm{TN}}\left(x\right)=\left\{\begin{array}{ll} \frac{\frac{1}{\sigma }\varphi \left(\frac{x-\mu }{\sigma }\right)}{\Phi \left(\frac{l_{U}-\mu }{\sigma }\right)-\Phi \left(\frac{l_{L}-\mu }{\sigma }\right)} & \text{if}\;l_{L}\leq x\leq l_{U},\\ 0 & \text{otherwise}, \end{array}\right.$$

where $\varphi \left(\cdot \right)$ and $\Phi \left(\cdot \right)$ are, respectively, the probability density function and the cumulative distribution function of the standard normal distribution. The estimates of parameters μ and σ are found as values which maximize the log‐likelihood function $l_{\mathrm{TN}}\left(\mu ,\sigma |x\right)$ of truncated normal distribution

$$\begin{array}{c} \begin{array}{ll} l_{\mathrm{TN}}\left(\mu ,\sigma |x\right)= & \;-\frac{n_{o}}{2}\log\left(2\pi \right)-n_{o}\log\left(\sigma \right)-\frac{1}{2}\sum_{i=1}^{n_{o}}{\left(\frac{x_{o,i}-\mu }{\sigma }\right)}^{2}-\\ & \;-n_{o}\log\left(\Phi \left(\frac{l_{U}-\mu }{\sigma }\right)-\Phi \left(\frac{l_{L}-\mu }{\sigma }\right)\right). \end{array} \end{array}$$

The truncated normal distribution fits the observations inside the measurable range with $\mathrm{TN}\left(\hat{\mu },{\hat{\sigma }}^{2},l_{L},l_{U}\right)$, however it is unclear, whether applying the estimates $\hat{\mu }$ and ${\hat{\sigma }}^{2}$ to the whole sample using $N\left(\hat{\mu },{\hat{\sigma }}^{2}\right)$ will produce a good fit.

### 2.4 Method 4: Using a Normal Distribution With Censored Observations

This approach takes into consideration the partial information we have about the truncated observations [12, 13, 18]. Let $n_{t,L}$ denote the number of observations that fall below $l_{L}$ and $n_{t,U}=n_{t}-n_{t,L}$ denote the number of observations that fall above $l_{U}$. The parameter estimates are then found as values which maximize the log‐likelihood function $l_{\mathrm{CN}}\left(\mu ,\sigma |x\right)$ of normal distribution with censored observations

$$\begin{array}{c} \begin{array}{ll} l_{\mathrm{CN}}\left(\mu ,\sigma |x\right)= & \;-\;\frac{n_{o}}{2}\log\left(2\pi \right)-n_{o}\log\left(\sigma \right)-\frac{1}{2}\sum_{i=1}^{n_{o}}{\left(\frac{x_{o,i}-\mu }{\sigma }\right)}^{2}+\\ & \;+\;n_{t,L}\log\left(\Phi \left(\frac{l_{L}-\mu }{\sigma }\right)\right)+n_{t,U}\log\left(1-\Phi \left(\frac{l_{U}-\mu }{\sigma }\right)\right). \end{array} \end{array}$$

Parameter estimates for Methods 3 and 4 cannot be expressed with explicit formulæ. Instead, we apply the Broyden‐Fletcher‐Goldfarb‐Shanno numerical method [20] to maximize $l_{\mathrm{TN}}\left(\mu ,\sigma |x\right)$ and $l_{\mathrm{CN}}\left(\mu ,\sigma |x\right)$ with respect to μ and σ.

In the following text, we denote Method 1 as 1 (*N* no), Method 2 with replacement by the limit as 2A (*N* $l_{L}/l_{U}$), Method 2 with replacement by $0.5l_{L}/1.5l_{U}$ as 2B (*N* $0.5l_{L}/1.5l_{U}$), Method 2 with replacement by $\sqrt{0.5}l_{L}/1+\sqrt{0.5}l_{U}$ as 2C (*N* $\sqrt{0.5}l_{L}/1+\sqrt{0.5}l_{U}$), Method 3 as 3 (TN), and Method 4 as 4 (CN).

## 3 Calculation

To compare the methods described in Section 2, we designed a simulation study. Then we applied the methods to real‐life data to show on specific examples, how the choice of method affects the results.

### 3.1 Simulation Study

The analyte is represented by a normally distributed random variable with mean μ=5 and standard deviation σ=2. Simulations were conducted for sample sizes n=10, 50, 100, 200, proportions of truncated observations p=0.05, 0.1, 0.15, 0.2, 0.4, 0.6 and number of runs of simulation M=1000.

The average relative error $\left(\hat{\mu }-\mu \right)/\mu$ and average ratio $\hat{\sigma }/\sigma$ for combinations of n and p are summarized in tables and visualized using line plots, where the proportion of truncated observation p is on the x‐axis and $\left(\hat{\mu }-\mu \right)/\mu$, or $\hat{\sigma }/\sigma$, is on the y‐axis, with the ideal situation $\left(\hat{\mu }-\mu \right)/\mu =0$, or $\hat{\sigma }/\sigma =1$, displayed as a horizontal line. Using $\left(\hat{\mu }-\mu \right)/\mu$ gives us not only information on the relative magnitude of the error but also the direction of the error, i.e. under‐ or overestimating μ. While the same measure can be used for σ, it is more illustrative to look at $\hat{\sigma }/\sigma$ to see how the spread of the distribution would be affected. MSEs of both parameters $\mathrm{MSE}\;\left(\hat{\mu }\right)$ and $\mathrm{MSE}\;\left(\hat{\sigma }\right)$ are summarized in tables.

$$\mathrm{MSE}\;\left(\hat{\mu }\right)=\frac{1}{M}\sum_{m=1}^{M}{\left(\hat{\mu }-\mu \right)}^{2},\;\mathrm{MSE}\;\left(\hat{\sigma }\right)=\frac{1}{M}\sum_{m=1}^{M}{\left(\hat{\sigma }-\sigma \right)}^{2}.$$

For *left‐sided truncation* (truncation below $l_{L}$), the detection limit was set to the p‐quantile of $N\left(5,4\right)$ This would correspond to 100×p % of the population falling below the detection limit. M=1000 random samples of length n from $N\left(5,4\right)$ were generated and any observation below the p‐quantile was removed from each sample. Using methods described in Section 2, estimates $\hat{\mu }$ and $\hat{\sigma }$ were calculated and compared to μ=5 and σ=2.

For *right‐sided truncation* (truncation above $l_{U}$), the detection limit was set to the $\left(1-p\right)$‐quantile of $N\left(5,4\right)$. This would correspond to 100×p % of the population falling above the detection limit. As with left‐sided truncation, M=1000 random samples of length n from $N\left(5,4\right)$ were generated, any observation above the $\left(1-p\right)$‐quantile was removed from each sample. Applying methods described in Section 2, estimates $\hat{\mu }$ and $\hat{\sigma }$ were calculated and compared to μ=5 and σ=2.

For *double‐sided truncation* (truncation below $l_{L}$ and above $l_{U}$), the lower detection limit was set to the $\left(p/2\right)$‐quantile and the upper detection limit was set to the $\left(1-p/2\right)$‐quantile of $N\left(5,4\right)$. This would correspond to symmetrical truncation with 100×p/2 % of the population falling below the lower detection limit and 100×p/2 % of the population falling above the upper detection limit, for a total of 100×p % outside the detection limits. M=1000 random samples of length n from $N\left(5,4\right)$ were generated, and any observation below the $\left(p/2\right)$‐quantile or above the $\left(1-p/2\right)$‐quantile was removed from each sample. Using methods described in Section 2, estimates $\hat{\mu }$ and $\hat{\sigma }$ were calculated and compared to μ=5 and σ=2.

### 3.2 Application to Real‐Life Data

Troponin T (TnT) is an important protein for proper contraction of the heart. The concentration of TnT in blood serum is measured to establish possible damage to the heart, for example, myocardial infarction or myocardial ischemia. Under normal circumstances, TnT is not present in the blood, but if the heart muscle is damaged, TnT is released into the blood. A sudden increase in TnT concentration is a sign of an acute problem, and stable concentrations of TnT in repeated measurements over a longer period of time indicate a chronic problem [21, 22, 23]. For this study, concentration of TnT in blood serum was measured using the Roche Cobas e411 analyzer, with LoB 3 ng/L.

D‐dimer (DDMPL) is a product of blood clot degradation. A high concentration of DDMPL in blood plasma may be a sign of hypercoagulation, which is associated with a higher risk of thrombosis [24, 25]. For this study, concentration of DDMPL was measured using the SYSMEX CA500 analyzer with MediRox D‐dimer kit MRX 147B, with LoD 0.3 mg/L FEU.

Human chorionic gonadotropin β (HCG) is a glycoprotein important for proper fetal development during pregnancy. HCG can be measured from urine or blood serum. It is used to confirm a pregnancy and to monitor its development. If HCG is present in a blood or urine sample, but pregnancy cannot be ascertained using ultrasound imaging, it might indicate an ectopic pregnancy. Apart from pregnancy, HCG can also indicate the presence of certain tumors, for example, testicular tumors, ovarian tumors, or choriocarcinoma, and may therefore be used to establish a cancer diagnosis and monitor the treatment [26, 27]. For this study, concentration of HCG in blood serum was measured using the Siemens Dimension Vista 1500 analyzer, with LoD 1 IU/L.

CRP indicates acute inflammation, but cannot point to a specific cause. CRP can be measured from blood, blood serum, or blood plasma [28, 29, 30]. For this study, concentration of CRP in blood serum was measured using the Siemens Dimension Vista 1500 analyzer, with LLoQ 2.9 mg/L.

Phospho‐tau pT217 (pT217) is a biomarker for Alzheimer's disease (AD). It has been shown that p‐tau T217 species (quantified as the pT217/T217 ratio) highly correlate with amyloid lesions in the brain, cognitive decline, and tau positron emission tomography (PET) imaging in AD [31]. For this study, pT217 concentrations in cerebrospinal fluid were measured using pT217 assay on the Quanterix Single molecule array (Simoa) HD‐1 Analyzer with LLoQ 184.4 pg/mL and LoD 102.2 pg/mL [32]. According to the manufacturer's recommendations, each measurement was performed in duplicate. Concentrations were calculated using Simoa HD‐1 instrument software.

Data on TNT, HCG, CRP, and DDMPL were measured at the SK‐Lab spol. s.r.o. laboratory in Lučenec (Slovakia) as part of a phase 1 clinical trial. Measurements were taken at the screening visit. Data on biomarker pT217 (n=125) were measured at the Institute of Neuroimmunology, Slovak Academy of Sciences (Bratislava, Slovakia) as part of a phase 2 clinical trial. Measurements were taken at one of the post‐baseline visits. Data were de‐identified, and all arms in each trial were joined for the purpose of this application. The data serve as illustration of the practical consequences of various aspects of the simulation study, that is, different sample sizes and various proportions of observations outside of measurable range.

## 4 Results

Results of simulation study, which was described in Section 3.1, are presented in Section 4.1 together with our recommendations regarding the choice of method in specified situations. Section 4.2 presents the results of the methods described in Section 2 applied to data described in Section 3.2.

### 4.1 Results of Simulation Study

**FIGURE 1:**
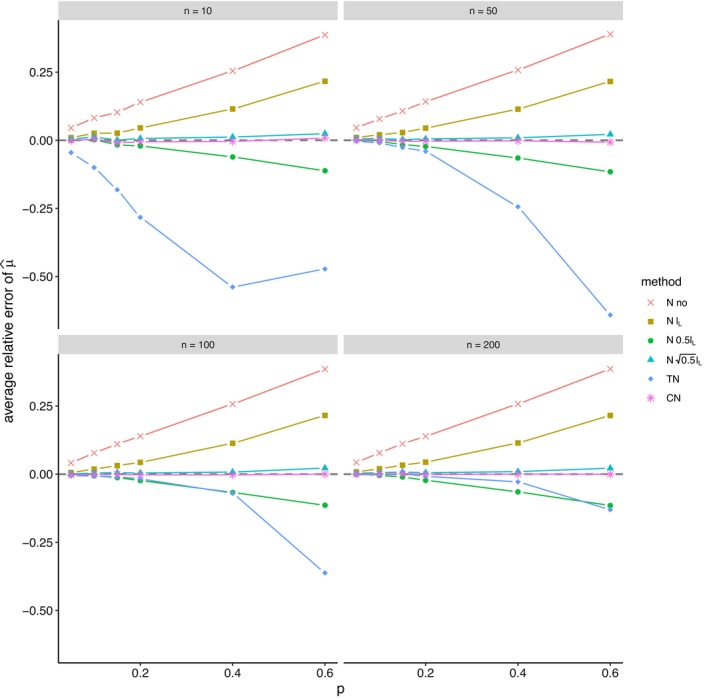
Average relative error of $\hat{\mu }$ —left‐sided truncation, Methods 1 (*N* no), 2A (*N* $l_{L}$), 2B (*N* $0.5l_{L}$), 2C (*N* $\sqrt{0.5}l_{L}$), 3 (TN), 4 (CN).

**FIGURE 2:**
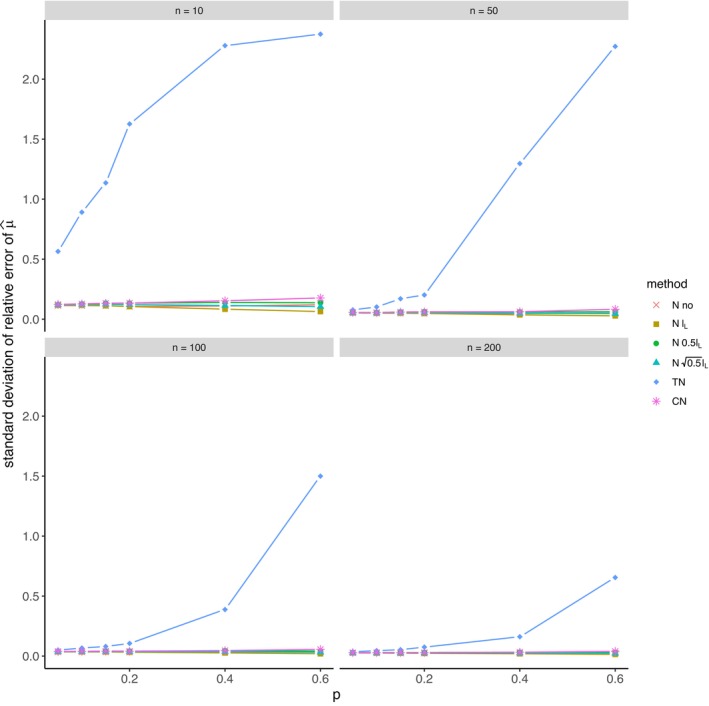
Standard deviation of relative error of $\hat{\mu }$ —left‐sided truncation, Methods 1 (*N* no), 2A (*N* $l_{L}$), 2B (*N* $0.5l_{L}$), 2C (*N* $\sqrt{0.5}l_{L}$), 3 (TN), 4 (CN).

**FIGURE 3:**
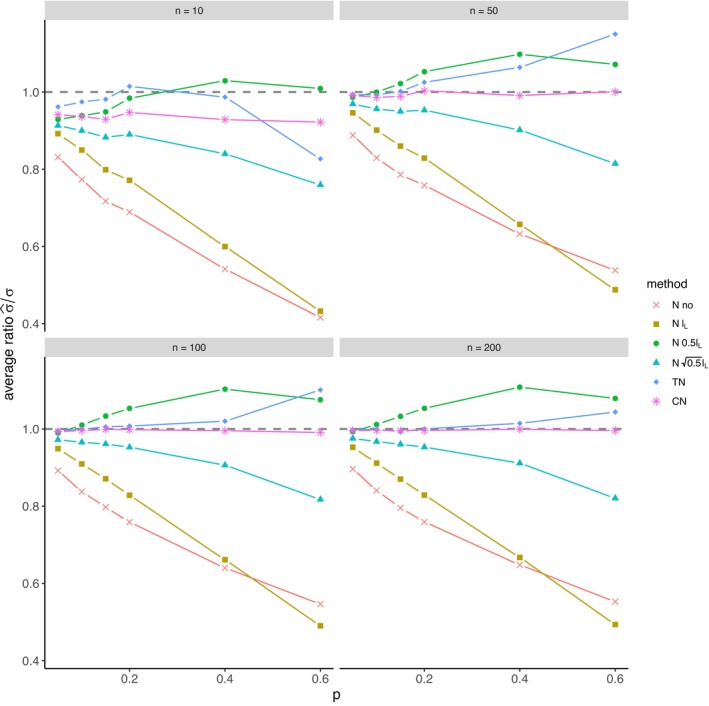
Average ratio $\frac{\hat{\sigma }}{\sigma }$ —left‐sided truncation, Methods 1 (*N* no), 2A (*N* $l_{L}$), 2B (*N* $0.5l_{L}$), 2C (*N* $\sqrt{0.5}l_{L}$), 3 (TN), 4 (CN).

**FIGURE 4:**
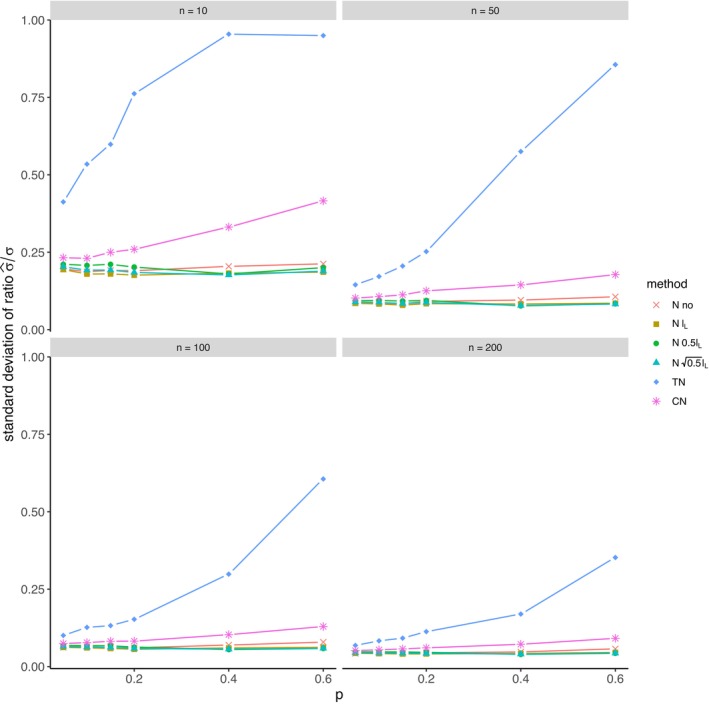
Standard deviation of ratio $\frac{\hat{\sigma }}{\sigma }$ —left‐sided truncation, Methods 1 (*N* no), 2A (*N* $l_{L}$), 2B (*N* $0.5l_{L}$), 2C (*N* $\sqrt{0.5}l_{L}$), 3 (TN), 4 (CN).

The average relative error of $\hat{\mu }$ and the average ratio $\hat{\sigma }/\sigma$ for *left‐sided truncation* are summarized in Tables A1 and A2 in Appendix A, respectively. Figures 1 and 2 display the average relative error of $\hat{\mu }$ and the standard deviation of relative error of $\hat{\mu }$, respectively. Figures 3 and 4 display the average ratio $\hat{\sigma }/\sigma$ and the standard deviation of ratio $\hat{\sigma }/\sigma$, respectively.

Methods 1 (*N* no) and 2A (*N* $l_{L}$) perform the worst across all sample sizes and proportions of truncated observations: Method 1 (*N* no) overestimates μ on average by 4% up to by $\left(39\%\right)$ and underestimates σ on average by 10% up to by 59%, Method 2A (*N* $l_{L}$) overestimates μ on average by 0.5% up to by 22% and underestimates σ on average by 5% up to by 57%.

Method 2B (*N* $0.5l_{L}$) provides estimates of μ for p≤0.1 on average with an error below 0.6%, and for p≥0.15 underestimates μ on average by 1% up to by 12% and underestimates σ on average by 1% up to by 10%.

Method 2C (*N* $\sqrt{0.5}l_{L}$) performs satisfactorily in estimating μ across all sample sizes and proportions of truncated observations. The estimate of σ is satisfactory up to p=0.2, although as p rises, σ is increasingly underestimated: μ is overestimated on average by less then 0.1% up to by 3%, while σ is underestimated on average by 2% up to by 24%.

Method 3 (TN) underestimates μ on average by less then 0.1% up to by 65% and estimates σ with various errors on average from underestimating by 17% to overestimating by 14%.

For n<50 and p≤0.4 Method 2B (*N* $0.5l_{L}$) produces a similar estimate of μ and better estimate of σ compared to Method 2C (*N* $\sqrt{0.5}l_{L}$). For n≥50 and p≤0.4, Methods 2B (*N* $0.5l_{L}$) and 3 (TN) produce similar average estimate of μ as Method 2C (*N* $\sqrt{0.5}l_{L}$), however the variances of estimates provided by Method 3 (TN) are greater than by any other method.

Method 4 (CN) produces the best estimates of both μ and σ across all sample sizes and proportions of truncated observations, except for the estimate of σ at n=10, where Method 2B (*N* $0.5l_{L}$) performs better: Method 4 (CN) provides estimates of μ on average with an error below 0.1% for any p and n, and provides estimates of σ on average with an error below 1.4% for n>10 and any p, for n=10 it underestimates σ on average by 6% up to by 8%.

MSE $\left(\hat{\mu }\right)$ and MSE $\left(\hat{\sigma }\right)$ are included in Tables A3 and A4 in Appendix A. Method 2C (*N* $\sqrt{0.5}l_{L}$) is the best based on MSE $\left(\hat{\mu }\right)$ and MSE $\left(\hat{\sigma }\right)$.

**FIGURE 5:**
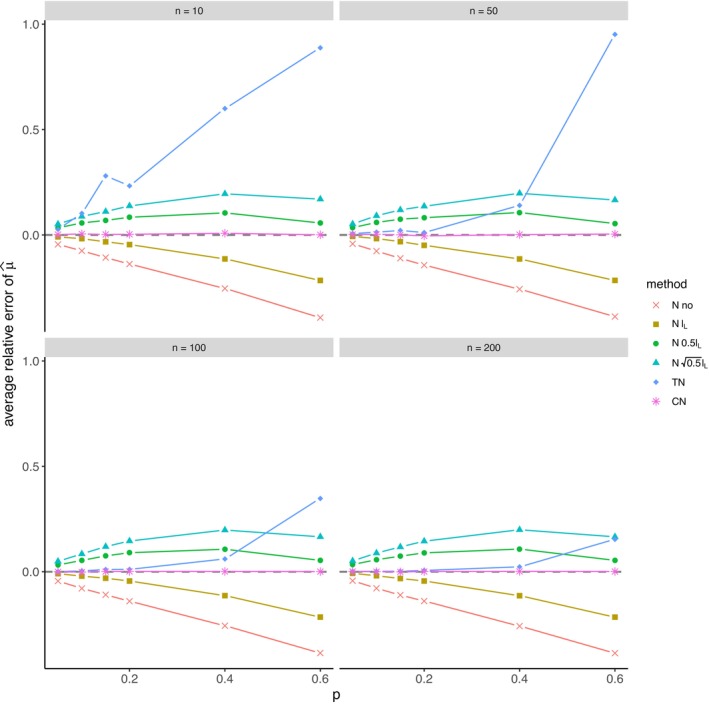
Average relative error of $\hat{\mu }$ —right‐sided truncation, Methods 1 (*N* no), 2A (*N* $l_{L}$), 2B (*N* $0.5l_{L}$), 2C (*N* $\sqrt{0.5}l_{L}$), 3 (TN), 4 (CN).

**FIGURE 6:**
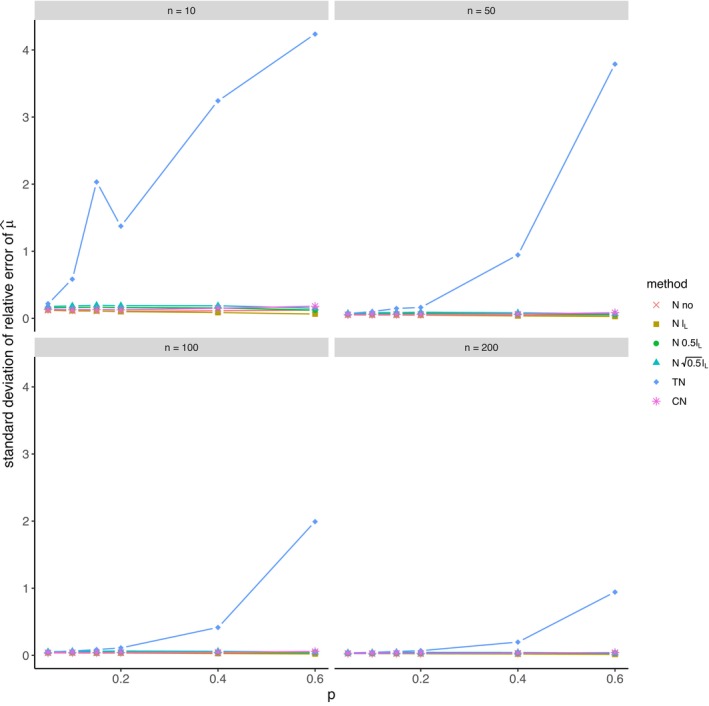
Standard deviation of relative error of $\hat{\mu }$ —right‐sided truncation, Methods 1 (*N* no), 2A (*N* $l_{L}$), 2B (*N* $0.5l_{L}$), 2C (*N* $\sqrt{0.5}l_{L}$), 3 (TN), 4 (CN).

**FIGURE 7:**
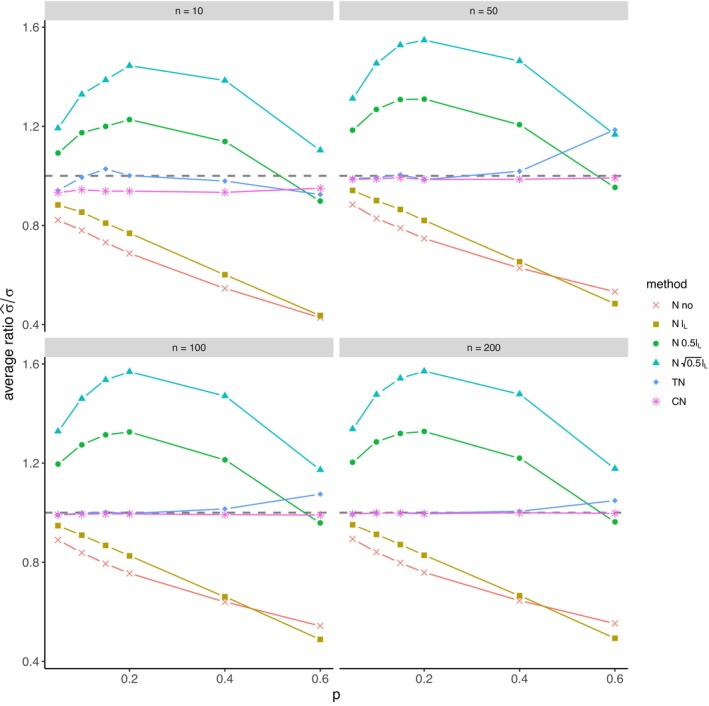
Average ratio $\frac{\hat{\sigma }}{\sigma }$ —right‐sided truncation, Methods 1 (*N* no), 2A (*N* $l_{L}$), 2B (*N* $0.5l_{L}$), 2C (*N* $\sqrt{0.5}l_{L}$), 3 (TN), 4 (CN).

**FIGURE 8:**
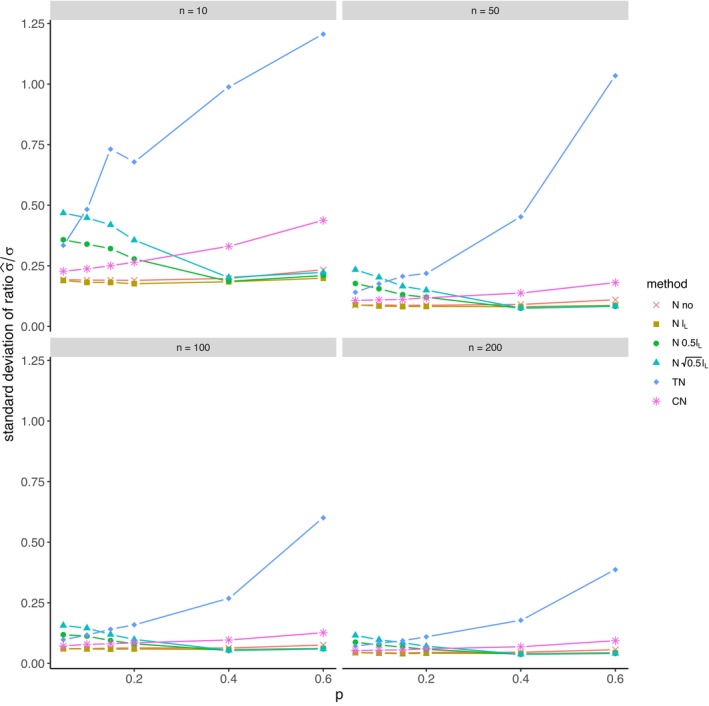
Standard deviation of ratio $\frac{\hat{\sigma }}{\sigma }$ —right‐sided truncation, Methods 1 (*N* no), 2A (*N* $l_{L}$), 2B (*N* $0.5l_{L}$), 2C (*N* $\sqrt{0.5}l_{L}$), 3 (TN), 4 (CN).

The average relative error of $\hat{\mu }$ and the average ratio $\hat{\sigma }/\sigma$ for *right‐sided truncation* are summarized in Tables B1 and B2 in Appendix B, respectively. Figures 5 and 6 display the average relative error of $\hat{\mu }$ and the standard deviation of relative error of $\hat{\mu }$, respectively. Figures 7 and 8 display the average ratio $\hat{\sigma }/\sigma$ and the standard deviation of ratio $\hat{\sigma }/\sigma$, respectively.

Methods 1 (*N* no) and 2A (*N* $l_{U}$) underestimate both μ and σ: Method 1 underestimates μ on average by 4% up to by 39% and σ on average by 11% up to by 57%, Method 2A underestimates μ on average by approx. 1% (for p=0.05 for any n) up to by 22% and σ on average by 5% up to by 56%.

Methods 2B (*N* $1.5l_{U}$) and 2C (*N* $\left(1+\sqrt{0.5}\right)l_{U}$) mostly overestimate both μ and σ: Method 2B overestimates μ on average by 3% up to by 11% and oberestimates σ on average by 9% up to by 33% for p≤0.4 and underestimates σ on average by 4% up to by 10% for p=0.6; Method 2C overestimates μ on average by 5% up to by 20% and σ on average by 19% up to by 57%.

Method 3 overestimates μ on average by less then 0.1% up to by 95% and estimates σ with various errors on average from underestimating by less then 0.1% to overestimating by 18%. For n≥50 and p≤0.4, average estimates by Method 3 (TN) seem satisfactory, but the variances of the estimates are large.

Method 4 (CN) produces the best estimates of both μ and σ across all sample sizes and proportions of truncated observations: Method 4 provides estimates of μ on average with an error below 0.1% and underestimates σ on average by less then 1.4% for n>10 and any p, for n=10 it underestimates σ on average by 5% up to by 7%.

MSE $\left(\hat{\mu }\right)$ and MSE $\left(\hat{\sigma }\right)$ are included in Tables B3 and B4 in Appendix B. Method 4 (CN) is the best based on MSE $\left(\hat{\mu }\right)$ and MSE $\left(\hat{\sigma }\right)$ as well.

**FIGURE 9:**
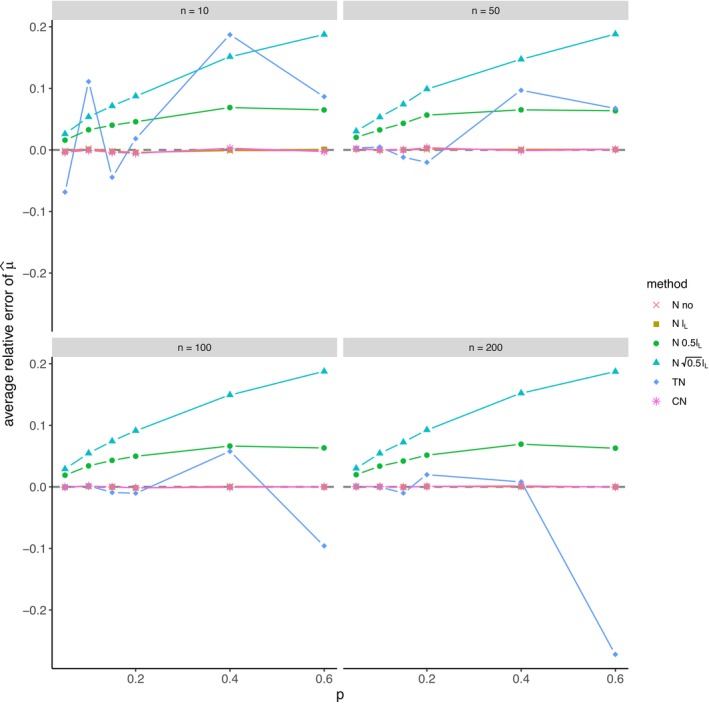
Average relative error of $\hat{\mu }$ —double‐sided truncation, Methods 1 (*N* no), 2A (*N* $l_{L}$), 2B (*N* $0.5l_{L}$), 2C (*N* $\sqrt{0.5}l_{L}$), 3 (TN), 4 (CN).

**FIGURE 10:**
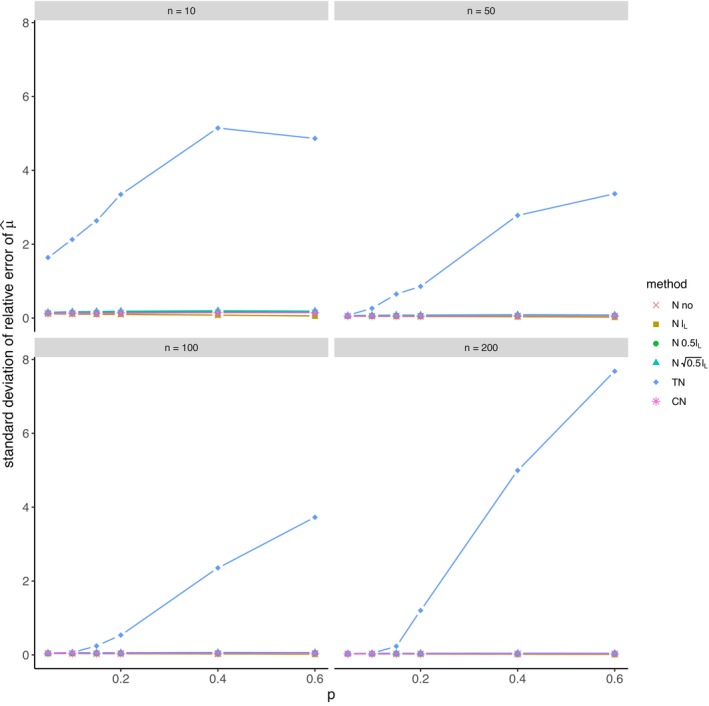
Standard deviation of relative error of $\hat{\mu }$ —double‐sided truncation, Methods 1 (*N* no), 2A (*N* $l_{L}$), 2B (*N* $0.5l_{L}$), 2C (*N* $\sqrt{0.5}l_{L}$), 3 (TN), 4 (CN).

**FIGURE 11:**
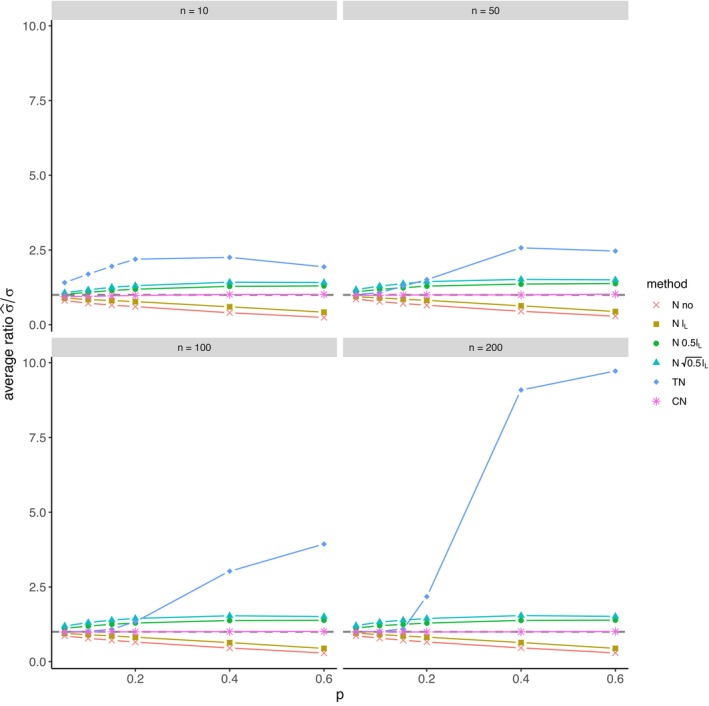
Average ratio $\frac{\hat{\sigma }}{\sigma }$ —double‐sided truncation, Methods 1 (*N* no), 2A (*N* $l_{L}$), 2B (*N* $0.5l_{L}$), 2C (*N* $\sqrt{0.5}l_{L}$), 3 (TN), 4 (CN).

**FIGURE 12:**
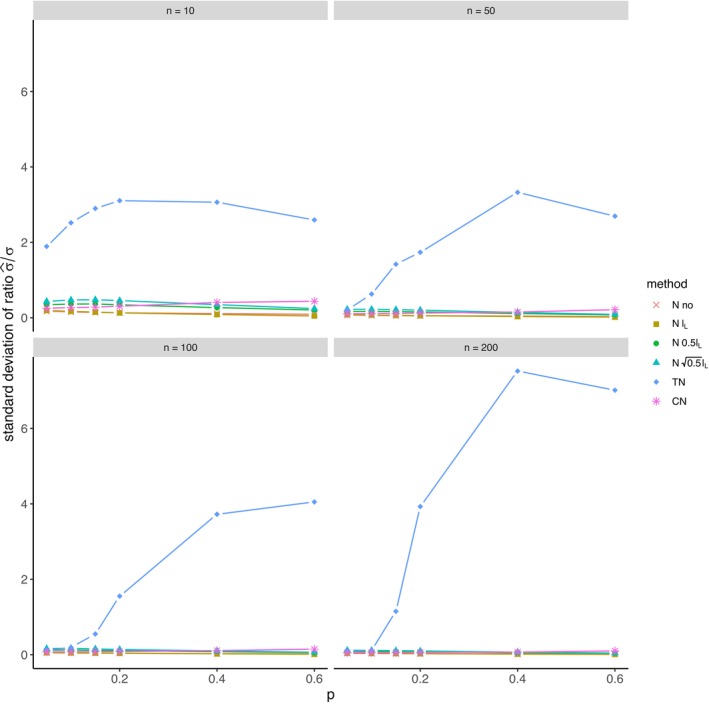
Standard deviation of ratio $\frac{\hat{\sigma }}{\sigma }$ —double‐sided truncation, Methods 1 (*N* no), 2A (*N* $l_{L}$), 2B (*N* $0.5l_{L}$), 2C (*N* $\sqrt{0.5}l_{L}$), 3 (TN), 4 (CN).

The average relative error of $\hat{\mu }$ and the average ratio $\hat{\sigma }/\sigma$ for *double‐sided truncation* are summarized in Tables C1 and C2 in Appendix C, respectively. Figures 9 and 10 display the average relative error of $\hat{\mu }$ and the standard deviation of relative error of $\hat{\mu }$, respectively. Figures 11 and 12 display the average ratio $\hat{\sigma }/\sigma$ and the standard deviation of ratio $\hat{\sigma }/\sigma$, respectively.

Symmetrical truncation removes approximately the same number of observations on each side, therefore it does not affect μ when using Methods 1 (*N* no) or 2A (*N* $l_{L}/l_{U}$) (because $|\mu -l_{L}|=|\mu -l_{U}|$), but they underestimate σ: Methods 1 and 2A provide estimates of μ on average with an error below 0.1% for any p and n, Method 1 underestimates σ on average by 13% up to by 76% and Method 2A underestimates σ on average by 5% up to by 58%.

Methods 2B (*N* $0.5l_{L}/1.5l_{U}$) and 2C (*N* $\sqrt{0.5}l_{L}/\left(1+\sqrt{0.5}\right)l_{U}$) overestimate both parameters: Method 2B overestimates μ on average by 2% (for p=0.05 for any n) up to by 7% and overestimates σ on average by 2% up to by 39%; Method 2C overestimates μ on average by 3% up to by 19% and overestimates σ on average by 7% up to by 54%.

Method 3 (TN) mostly overestimates the parameters and the variances of the estimates are large: Method 3 estimates μ with various errors on average from underestimating by less then 0.1% to overestimating by 27%, and overestimates σ on average by less then 1% up to by 872%.

Method 4 (CN) produces the best estimates of both μ and σ across all sample sizes and proportions of truncated observations: Method 4 provides estimates of μ on average with an error below 0.6% for any p and n and estimates of σ on average with an error below 1.5% for n>10 and p≤0.4, for p=0.6 the error is on average below 2.4%, for n=10 the error is on average between 1% and 5%.

MSE $\left(\hat{\mu }\right)$ and MSE $\left(\hat{\sigma }\right)$ are included in Tables C3 and C4 in Appendix C. While Methods 1 (*N* no) and 2A (*N* $l_{L}/l_{U}$) have the lowest MSE of $\hat{\mu }$, it is again due to symmetrical truncation. Method 4 (CN) is the best based on MSE $\left(\hat{\mu }\right)$ and MSE $\left(\hat{\sigma }\right)$.

Based on the results for all three truncation options, we do not recommend working with samples whose proportion of truncated observations reaches 0.4 or more. We also recommend extreme caution if the sample size is small, even with lower proportions of truncated observations. Although measurements of analytes are always non‐negative, the assumed normal distribution has no lower (or upper) limit, and therefore no restriction is placed on the possible values of μ. Methods 1 and 2 will always lead to non‐negative estimates of μ; however Method 3 may lead to negative estimates of μ; Method 4 may produce negative estimates of μ for samples with a high proportion of truncated observations. Table D1 in Appendix D provides overview of the overall performance of the methods for all three truncation options based on $\left(\hat{\mu }-\mu \right)/\mu$, $\hat{\sigma }/\sigma$, MSE $\left(\hat{\mu }\right)$ and MSE $\left(\hat{\sigma }\right)$.

### 4.2 Application to Real‐Life Data

Table 1 summarizes the results for the data described in Section 3.2. CRP has a proportion of truncated observations above 0.8, and therefore we would not recommend working with these data at all. HCG has a proportion of truncated observations above 0.4, and we would still caution against working with these data, however if one would have to use them, Method 4 would be the best to use based on the results of the simulation study. For TnT, DDMPL and pT217 we would recommend Method 4. Only one TnT observation is below the limit, which explains why Method 1, Method 2 with any of the three replacement options and Method 4 produce very similar results.

**TABLE 1:** Parameter estimates, Methods 1 (*N* no), 2A (*N* $l_{L}$), 2B (*N* $0.5l_{L}$), 2C (*N* $\sqrt{0.5}l_{L}$), 3 (TN), 4 (CN).

Variable	Method	n	p	$\hat{\mu }$	$\hat{\sigma }$
TnT	1	33	0.0303	6.1250	2.9448
TnT	2A	33	0.0303	6.0303	2.9489
TnT	2B	33	0.0303	5.9848	3.0063
TnT	2C	33	0.0303	6.0037	2.9800
TnT	3	33	0.0303	−26.0646	10.4491
TnT	4	33	0.0303	5.9821	3.0194
DDMPL	1	33	0.2727	0.4958	0.2189
DDMPL	2A	33	0.2727	0.4424	0.2060
DDMPL	2B	33	0.2727	0.4015	0.2420
DDMPL	2C	33	0.2727	0.4185	0.2254
DDMPL	3	33	0.2727	−8.9679	1.3766
DDMPL	4	33	0.2727	0.3941	0.2604
HCG	1	33	0.4545	2.8889	1.6630
HCG	2A	33	0.4545	2.0303	1.5469
HCG	2B	33	0.4545	1.8030	1.7098
HCG	2C	33	0.4545	1.8972	1.6397
HCG	3	33	0.4545	−5.1982	4.2471
HCG	4	33	0.4545	1.1676	2.4529
CRP	1	33	0.8182	7.8667	3.7650
CRP	2A	33	0.8182	3.8030	2.4994
CRP	2B	33	0.8182	2.6167	2.9500
CRP	2C	33	0.8182	3.1081	2.7585
CRP	3	33	0.8182	2.7635	6.2863
CRP	4	33	0.8182	−5.1006	8.8630
pT217	1	125	0.2400	456.9518	218.8278
pT217	2A	125	0.2400	391.5394	223.4783
pT217	2B	125	0.2400	369.4114	246.2931
pT217	2C	125	0.2400	378.5771	236.3147
pT217	3	125	0.2400	129.6228	369.1371
pT217	4	125	0.2400	350.5646	277.2552

## 5 Discussion

When dealing with data subject to measurement limits, the choice of method of estimating the mean and the standard deviation is important. It may be tempting to use methods that are easy to implement, however selecting an incorrect method may lead to biased estimates and change the research outcomes, e.g., incorrect result of two‐sample test about means when comparing two populations or biased estimation of regression line. Using a simulation study we compared four methods by looking at relative error $\frac{\hat{\mu }-\mu }{\mu }$, ratio $\frac{\hat{\sigma }}{\sigma }$, and MSE $\left(\hat{\mu }\right)$ and MSE $\left(\hat{\sigma }\right)$. Based on the results of this simulation study, if the amount of observations outside of measurable range is below 40%, we recommend using a normal distribution with censored observations to estimate mean and standard deviation when dealing with normally distributed data subject to measurement limits in practice. The second best option for left‐sided truncation would be replacement by 0.5‐times the limit, for right‐sided and symmetric double‐sided truncation it would be replacement by the limit. If the amount of observations outside of measurable range exceeds 40%, we advise not to use the data for any statistical analysis. These results are important not only for researchers but should be incorporated into guidelines for good statistical practice in clinical trials to raise awareness of the way data subject to measurement limits should be handled.

To illustrate how the choice of method can affect the estimates, we applied the methods to real‐life laboratory data with various proportions of truncation and sample sizes. All of the data were subject to lower limits, but not to upper limits. TnT, DDMPL, HCG, and CRP had 33 observations each and pT217 had 125 observations. We do not recommend working with data on CRP, as it has over 80% of observations below lower limit. HCG has 45% of observations below lower limit and we would still caution against working with these data, but if necessary using normal distribution with censored observations would be the best choice. TnT, DDMPL, and pT217 have 3%, 27%, and 24% of observations below lower limit, respectively, which means normal distribution with censored observations should be used for the estimation of mean and standard deviation.

## Author Contributions

M.J. and S.K. conceptualized the simulation study, M.J. wrote the main manuscript text, performed the simulation study and data analysis, and prepared tables, S.K. supervised the simulation study and prepared figures, J.H. provided data and domain expertise. All authors reviewed the manuscript.

## Conflicts of Interest

The authors declare no conflicts of interest.

## Supporting information


Appendix S1.

## Data Availability

Data from simulation study available on request. Data from clinical trials not available due to legal restrictions.
