# Supplementary material for: Are We Estimating the Mean and Variance Correctly in the Presence of Observations Outside of Measurable Range?
Source: Pharmacol Res Perspect. 2024 Dec 20;13(1):e70048. doi: 10.1002/prp2.70048 (PMC11661985; doi:10.1002/prp2.70048)
Supplement: Supplementary file 1 — Appendix S1. [file PRP2-13-e70048-s001.pdf]

Are We Estimating the Mean and Variance Correctly in the Presence of Observations Outside of Measurable Range?

Markéta Janošová*[1], Stanislav Katina†[1], and Jozef Hanes‡[2]

[1]Department of Mathematics and Statistics, Faculty of Science, Masaryk University, Brno, Czech Republic
[2]Institute of Neuroimmunology, Slovak Academy of Sciences, Bratislava, Slovakia

Appendix A Results of simulation study for left-sided truncation

*janosova@math.muni.cz
†katina@math.muni.cz
‡jozef.hanes@savba.sk

Table A1: Average relative error of $\hat{\mu}$ – left-sided truncation, Methods 1 (N no), 2A (N l_L), 2B (N $0.5l_L$), 2C (N $\sqrt{0.5}l_L$), 3 (TN), 4 (CN)

method	n	$p = 0.05$	$p = 0.1$	$p = 0.15$	$p = 0.2$	$p = 0.4$	$p = 0.6$
1	10	0.0453	0.0821	0.1024	0.1401	0.2544	0.3863
2A	10	0.0095	0.0258	0.0263	0.0454	0.1149	0.2164
2B	10	0.0008	0.0027	-0.0170	-0.0208	-0.0610	-0.1118
2C	10	0.0044	0.0122	0.0009	0.0066	0.0118	0.0242
3	10	-0.0451	-0.0998	-0.1813	-0.2826	-0.5385	-0.4720
4	10	-0.0013	0.0047	-0.0092	-0.0056	-0.0041	0.0082
1	50	0.0460	0.0784	0.1073	0.1420	0.2579	0.3892
2A	50	0.0105	0.0202	0.0285	0.0447	0.1143	0.2159
2B	50	0.0018	-0.0037	-0.0157	-0.0228	-0.0651	-0.1157
2C	50	0.0054	0.0062	0.0026	0.0051	0.0092	0.0216
3	50	-0.0034	-0.0098	-0.0265	-0.0404	-0.2438	-0.6408
4	50	0.0015	0.0010	-0.0040	-0.0033	-0.0019	-0.0068
1	100	0.0412	0.0779	0.1103	0.1393	0.2573	0.3853
2A	100	0.0060	0.0186	0.0314	0.0434	0.1133	0.2154
2B	100	-0.0026	-0.0059	-0.0126	-0.0234	-0.0670	-0.1141
2C	100	0.0009	0.0042	0.0056	0.0043	0.0077	0.0224
3	100	-0.0054	-0.0066	-0.0096	-0.0173	-0.0697	-0.3620
4	100	-0.0027	-0.0009	-0.0005	-0.0025	-0.0025	0.0002
1	200	0.0432	0.0782	0.1116	0.1392	0.2580	0.3858
2A	200	0.0084	0.0196	0.0332	0.0441	0.1145	0.2154
2B	200	-0.0000	-0.0045	-0.0104	-0.0223	-0.0649	-0.1147
2C	200	0.0035	0.0055	0.0077	0.0052	0.0094	0.0220
3	200	-0.0016	-0.0024	0.0016	-0.0080	-0.0280	-0.1302
4	200	0.0000	0.0007	0.0022	-0.0009	-0.0000	-0.0000

Table A2: Average ratio $\frac{\hat{\sigma}}{\sigma}$ – left-sided truncation, Methods 1 (N no), 2A (N l_L), 2B (N $0.5l_L$), 2C (N $\sqrt{0.5}l_L$), 3 (TN), 4 (CN)

method	n	$p = 0.05$	$p = 0.1$	$p = 0.15$	$p = 0.2$	$p = 0.4$	$p = 0.6$
1	10	0.8313	0.7733	0.7174	0.6889	0.5414	0.4161
2A	10	0.8921	0.8498	0.7988	0.7713	0.5997	0.4326
2B	10	0.9291	0.9386	0.9487	0.9839	1.0294	1.0093
2C	10	0.9132	0.8999	0.8828	0.8900	0.8400	0.7594
3	10	0.9616	0.9744	0.9813	1.0147	0.9868	0.8270
4	10	0.9413	0.9364	0.9296	0.9470	0.9285	0.9221
1	50	0.8881	0.8295	0.7858	0.7583	0.6324	0.5381
2A	50	0.9462	0.9013	0.8600	0.8288	0.6573	0.4878
2B	50	0.9865	0.9997	1.0219	1.0530	1.0977	1.0717
2C	50	0.9690	0.9562	0.9498	0.9529	0.9016	0.8146
3	50	0.9937	0.9922	1.0017	1.0252	1.0640	1.1500
4	50	0.9912	0.9860	0.9888	1.0033	0.9911	1.0008
1	100	0.8921	0.8376	0.7974	0.7587	0.6400	0.5465
2A	100	0.9491	0.9094	0.8710	0.8283	0.6613	0.4903
2B	100	0.9897	1.0101	1.0335	1.0533	1.1032	1.0758
2C	100	0.9720	0.9656	0.9611	0.9527	0.9063	0.8174
3	100	0.9958	0.9992	1.0056	1.0076	1.0203	1.1012
4	100	0.9933	0.9956	0.9989	0.9981	0.9955	0.9909
1	200	0.8964	0.8408	0.7957	0.7593	0.6480	0.5527
2A	200	0.9527	0.9116	0.8702	0.8288	0.6672	0.4935
2B	200	0.9930	1.0116	1.0329	1.0535	1.1082	1.0792
2C	200	0.9755	0.9673	0.9604	0.9530	0.9115	0.8206
3	200	0.9980	0.9990	0.9929	1.0003	1.0146	1.0438
4	200	0.9959	0.9962	0.9959	0.9963	0.9993	0.9957

Table A3: MSE($\hat{\mu}$) – left-sided truncation, methods 1 (N no), 2A (N l_L), 2B (N $0.5l_L$), 2C (N $\sqrt{0.5}l_L$), 3 (TN), 4 (CN)

method	n	$p = 0.05$	$p = 0.1$	$p = 0.15$	$p = 0.2$	$p = 0.4$	$p = 0.6$
1	10	0.3739	0.4887	0.5769	0.7723	1.9190	4.0906
2A	10	0.3413	0.3501	0.3301	0.3267	0.5033	1.2718
2B	10	0.3676	0.4052	0.4476	0.4589	0.5738	0.7790
2C	10	0.3555	0.3762	0.3808	0.3672	0.3278	0.2853
3	10	8.0048	20.0916	33.0283	68.0936	137.0297	146.4751
4	10	0.3783	0.4045	0.4405	0.4492	0.5869	0.7800
1	50	0.1190	0.2170	0.3490	0.5636	1.7185	3.8480
2A	50	0.0731	0.0731	0.0825	0.1065	0.3583	1.1843
2B	50	0.0766	0.0765	0.0946	0.1065	0.1945	0.4284
2C	50	0.0746	0.0710	0.0764	0.0766	0.0616	0.0657
3	50	0.1492	0.2605	0.7401	1.0558	43.4788	139.2836
4	50	0.0781	0.0753	0.0871	0.0922	0.0968	0.1697
1	100	0.0733	0.1822	0.3345	0.5140	1.6838	3.7430
2A	100	0.0343	0.0402	0.0555	0.0738	0.3391	1.1693
2B	100	0.0367	0.0397	0.0475	0.0564	0.1620	0.3696
2C	100	0.0352	0.0360	0.0384	0.0355	0.0351	0.0382
3	100	0.0619	0.1137	0.1630	0.2877	3.8844	59.4432
4	100	0.0373	0.0385	0.0421	0.0410	0.0534	0.0776
1	200	0.0640	0.1692	0.3260	0.4991	1.6788	3.7377
2A	200	0.0205	0.0271	0.0431	0.0623	0.3375	1.1646
2B	200	0.0204	0.0220	0.0248	0.0350	0.1306	0.3520
2C	200	0.0199	0.0204	0.0205	0.0190	0.0196	0.0258
3	200	0.0325	0.0514	0.0714	0.1415	0.6689	11.1336
4	200	0.0206	0.0213	0.0216	0.0218	0.0265	0.0390

Table A4: MSE($\hat{\sigma}$) – left-sided truncation, methods 1 (N no), 2A (N l_L), 2B (N $0.5l_L$), 2C (N $\sqrt{0.5}l_L$), 3 (TN), 4 (CN)

method	n	$p = 0.05$	$p = 0.1$	$p = 0.15$	$p = 0.2$	$p = 0.4$	$p = 0.6$
1	10	0.2673	0.3455	0.4657	0.5320	1.0081	1.5439
2A	10	0.1972	0.2191	0.2914	0.3330	0.7738	1.4254
2B	10	0.1982	0.1870	0.1883	0.1640	0.1328	0.1604
2C	10	0.1944	0.1877	0.2029	0.1846	0.2269	0.3742
3	10	0.6859	1.1444	1.4329	2.3214	3.6400	3.7231
4	10	0.2292	0.2282	0.2693	0.2803	0.4589	0.7151
1	50	0.0799	0.1457	0.2117	0.2668	0.5771	0.8985
2A	50	0.0405	0.0665	0.1035	0.1454	0.4971	1.0785
2B	50	0.0354	0.0354	0.0359	0.0467	0.0617	0.0483
2C	50	0.0356	0.0386	0.0386	0.0395	0.0630	0.1647
3	50	0.0840	0.1179	0.1687	0.2566	1.3387	3.0163
4	50	0.0419	0.0462	0.0509	0.0628	0.0836	0.1262
1	100	0.0619	0.1220	0.1792	0.2476	0.5378	0.8475
2A	100	0.0258	0.0474	0.0803	0.1304	0.4733	1.0548
2B	100	0.0190	0.0187	0.0225	0.0271	0.0546	0.0366
2C	100	0.0201	0.0209	0.0215	0.0226	0.0478	0.1473
3	100	0.0405	0.0641	0.0700	0.0935	0.3578	1.5078
4	100	0.0223	0.0240	0.0267	0.0270	0.0425	0.0671
1	200	0.0502	0.1090	0.1744	0.2397	0.5046	0.8133
2A	200	0.0163	0.0383	0.0741	0.1239	0.4503	1.0344
2B	200	0.0092	0.0096	0.0133	0.0199	0.0530	0.0324
2C	200	0.0106	0.0122	0.0138	0.0161	0.0378	0.1362
3	200	0.0187	0.0276	0.0339	0.0510	0.1159	0.5035
4	200	0.0107	0.0118	0.0131	0.0147	0.0208	0.0333

Appendix B Results of simulation study for right-sided truncation

Table B1: Average relative error of $\hat{\mu}$ – right-sided truncation, Methods 1 (N no), 2A (N l_U), 2B (N $1.5 l_U$), 2C (N $(1+\sqrt{0.5})l_U$), 3 (TN), 4 (CN)

method	n	$p = 0.05$	$p = 0.1$	$p = 0.15$	$p = 0.2$	$p = 0.4$	$p = 0.6$
1	10	-0.0448	-0.0753	-0.1075	-0.1381	-0.2540	-0.3922
2A	10	-0.0091	-0.0177	-0.0326	-0.0457	-0.1137	-0.2158
2B	10	0.0336	0.0570	0.0690	0.0840	0.1046	0.0569
2C	10	0.0513	0.0879	0.1110	0.1377	0.1950	0.1699
3	10	0.0264	0.1024	0.2801	0.2329	0.5997	0.8877
4	10	0.0019	0.0048	0.0023	0.0032	0.0078	0.0007
1	50	-0.0421	-0.0766	-0.1107	-0.1433	-0.2572	-0.3872
2A	50	-0.0069	-0.0171	-0.0317	-0.0492	-0.1139	-0.2156
2B	50	0.0348	0.0593	0.0747	0.0819	0.1062	0.0542
2C	50	0.0520	0.0910	0.1188	0.1362	0.1974	0.1659
3	50	0.0065	0.0131	0.0213	0.0115	0.1401	0.9513
4	50	0.0020	0.0029	0.0006	-0.0046	0.0017	0.0042
1	100	-0.0442	-0.0790	-0.1092	-0.1399	-0.2563	-0.3856
2A	100	-0.0091	-0.0207	-0.0307	-0.0441	-0.1134	-0.2152
2B	100	0.0323	0.0537	0.0753	0.0903	0.1066	0.0542
2C	100	0.0494	0.0846	0.1193	0.1460	0.1978	0.1658
3	100	0.0004	0.0041	0.0106	0.0114	0.0607	0.3476
4	100	-0.0006	-0.0017	0.0009	0.0015	0.0013	0.0005
1	200	-0.0427	-0.0784	-0.1108	-0.1391	-0.2579	-0.3861
2A	200	-0.0075	-0.0189	-0.0321	-0.0440	-0.1136	-0.2153
2B	200	0.0342	0.0571	0.0738	0.0897	0.1075	0.0542
2C	200	0.0514	0.0886	0.1176	0.1450	0.1991	0.1658
3	200	0.0015	0.0020	0.0023	0.0063	0.0233	0.1537
4	200	0.0010	0.0003	-0.0008	0.0009	0.0018	0.0010

Table B2: Average ratio $\frac{\hat{\sigma}}{\sigma}$ – right-sided truncation, Methods 1 (N no), 2A (N l_U), 2B (N $1.5l_U$), 2C (N $(1+\sqrt{0.5})l_U$), 3 (TN), 4 (CN)

method	n	$p = 0.05$	$p = 0.1$	$p = 0.15$	$p = 0.2$	$p = 0.4$	$p = 0.6$
1	10	0.8216	0.7804	0.7315	0.6872	0.5461	0.4282
2A	10	0.8828	0.8537	0.8094	0.7685	0.6011	0.4367
2B	10	1.0923	1.1743	1.1997	1.2267	1.1387	0.8986
2C	10	1.1925	1.3286	1.3867	1.4444	1.3848	1.1035
3	10	0.9408	0.9941	1.0279	1.0010	0.9793	0.9248
4	10	0.9323	0.9441	0.9382	0.9385	0.9336	0.9499
1	50	0.8843	0.8281	0.7893	0.7471	0.6282	0.5329
2A	50	0.9414	0.9005	0.8644	0.8202	0.6539	0.4846
2B	50	1.1846	1.2678	1.3081	1.3097	1.2064	0.9536
2C	50	1.3120	1.4539	1.5277	1.5476	1.4635	1.1670
3	50	0.9892	0.9928	1.0044	0.9851	1.0186	1.1857
4	50	0.9861	0.9880	0.9930	0.9856	0.9863	0.9916
1	100	0.8903	0.8384	0.7944	0.7550	0.6399	0.5434
2A	100	0.9476	0.9091	0.8681	0.8258	0.6607	0.4887
2B	100	1.1963	1.2738	1.3142	1.3252	1.2132	0.9586
2C	100	1.3282	1.4600	1.5357	1.5683	1.4710	1.1731
3	100	0.9903	0.9984	1.0017	0.9967	1.0152	1.0746
4	100	0.9913	0.9937	0.9951	0.9947	0.9921	0.9904
1	200	0.8940	0.8408	0.7975	0.7586	0.6453	0.5530
2A	200	0.9513	0.9125	0.8716	0.8283	0.6652	0.4936
2B	200	1.2033	1.2858	1.3195	1.3273	1.2195	0.9629
2C	200	1.3376	1.4764	1.5421	1.5706	1.4782	1.1776
3	200	0.9944	0.9986	0.9989	0.9979	1.0056	1.0486
4	200	0.9950	0.9985	0.9982	0.9956	0.9996	0.9974

Table B3: MSE($\hat{\mu}$) – right-sided truncation, methods 1 (N no), 2A (N l_U), 2B (N $1.5l_U$), 2C (N $(1+\sqrt{0.5})l_U$), 3 (TN), 4 (CN)

method	n	$p = 0.05$	$p = 0.1$	$p = 0.15$	$p = 0.2$	$p = 0.4$	$p = 0.6$
1	10	0.4112	0.4464	0.5828	0.7536	1.9301	4.2262
2A	10	0.3980	0.3358	0.3292	0.3070	0.5092	1.2688
2B	10	0.6654	0.7314	0.8129	0.8155	0.8760	0.4655
2C	10	0.8520	1.0475	1.2567	1.3653	1.8296	1.2937
3	10	1.2144	8.7706	105.1113	48.5308	271.4196	467.7347
4	10	0.4465	0.4085	0.4311	0.4046	0.5669	0.8119
1	50	0.1090	0.2102	0.3691	0.5742	1.7082	3.8106
2A	50	0.0697	0.0758	0.0880	0.1195	0.3588	1.1816
2B	50	0.1412	0.2262	0.2788	0.3163	0.3975	0.1495
2C	50	0.2058	0.3898	0.5415	0.6706	1.1438	0.8023
3	50	0.1293	0.2583	0.5561	0.6653	22.7746	381.0412
4	50	0.0760	0.0849	0.0854	0.0899	0.1008	0.1779
1	100	0.0800	0.1878	0.3260	0.5197	1.6690	3.7499
2A	100	0.0364	0.0441	0.0524	0.0782	0.3388	1.1681
2B	100	0.0820	0.1392	0.2099	0.2763	0.3435	0.1103
2C	100	0.1306	0.2677	0.4490	0.6329	1.0651	0.7421
3	100	0.0585	0.1040	0.1810	0.3101	4.3889	102.0695
4	100	0.0380	0.0410	0.0403	0.0447	0.0511	0.0787
1	200	0.0623	0.1691	0.3222	0.4980	1.6763	3.7433
2A	200	0.0194	0.0250	0.0416	0.0615	0.3316	1.1634
2B	200	0.0586	0.1130	0.1725	0.2342	0.3191	0.0917
2C	200	0.1027	0.2378	0.3957	0.5721	1.0355	0.7148
3	200	0.0307	0.0503	0.0818	0.1200	0.9796	22.7513
4	200	0.0200	0.0194	0.0217	0.0199	0.0263	0.0433

Table B4: MSE($\hat{\sigma}$) – right-sided truncation, methods 1 (N no), 2A (N l_U), 2B (N $1.5 l_U$), 2C (N $(1+\sqrt{0.5}) l_U$), 3 (TN), 4 (CN)

method	n	$p = 0.05$	$p = 0.1$	$p = 0.15$	$p = 0.2$	$p = 0.4$	$p = 0.6$
1	10	0.2762	0.3386	0.4337	0.5356	0.9809	1.5257
2A	10	0.1983	0.2174	0.2767	0.3384	0.7718	1.4268
2B	10	0.5442	0.5817	0.5720	0.5152	0.2153	0.2166
2C	10	1.0225	1.2349	1.2998	1.2958	0.7558	0.2411
3	10	0.4607	0.9316	2.1407	1.8409	3.9029	5.8351
4	10	0.2252	0.2377	0.2660	0.2954	0.4539	0.7750
1	50	0.0841	0.1494	0.2079	0.2863	0.5859	0.9210
2A	50	0.0454	0.0677	0.1002	0.1564	0.5056	1.0926
2B	50	0.2616	0.3831	0.4481	0.4412	0.1929	0.0360
2C	50	0.6080	0.9879	1.2236	1.2884	0.8829	0.1398
3	50	0.0796	0.1237	0.1706	0.1921	0.8184	4.4127
4	50	0.0465	0.0483	0.0498	0.0564	0.0764	0.1306
1	100	0.0629	0.1193	0.1848	0.2563	0.5347	0.8568
2A	100	0.0255	0.0472	0.0832	0.1355	0.4734	1.0611
2B	100	0.2099	0.3503	0.4301	0.4492	0.1929	0.0210
2C	100	0.5282	0.9307	1.2044	1.3306	0.8991	0.1342
3	100	0.0382	0.0545	0.0789	0.1010	0.2877	1.4636
4	100	0.0208	0.0247	0.0260	0.0287	0.0373	0.0640
1	200	0.0529	0.1089	0.1711	0.2412	0.5115	0.8119
2A	200	0.0173	0.0376	0.0723	0.1249	0.4548	1.0335
2B	200	0.1958	0.3496	0.4262	0.4419	0.1981	0.0122
2C	200	0.5090	0.9455	1.2043	1.3222	0.9204	0.1329
3	200	0.0203	0.0275	0.0347	0.0480	0.1261	0.6067
4	200	0.0112	0.0118	0.0124	0.0144	0.0187	0.0348

Appendix C Results of simulation study for double-sided truncation

Table C1: Average relative error of $\hat{\mu}$ – double-sided truncation, Methods 1 (N no), 2A (N l_L/l_U), 2B (N $0.5l_L/1.5l_U$), 2C (N $\sqrt{0.5}l_L/(1+\sqrt{0.5})l_U$), 3 (TN), 4 (CN)

method	n	$p = 0.05$	$p = 0.1$	$p = 0.15$	$p = 0.2$	$p = 0.4$	$p = 0.6$
1	10	-0.0015	0.0023	-0.0019	-0.0040	-0.0012	-0.0010
2A	10	-0.0031	0.0002	-0.0029	-0.0042	-0.0001	0.0009
2B	10	0.0158	0.0327	0.0400	0.0458	0.0688	0.0651
2C	10	0.0260	0.0536	0.0714	0.0874	0.1514	0.1875
3	10	-0.0685	0.1111	-0.0444	0.0183	0.1870	0.0865
4	10	-0.0036	-0.0007	-0.0038	-0.0054	0.0028	-0.0027
1	50	0.0024	0.0003	0.0003	0.0008	0.0011	0.0011
2A	50	0.0017	-0.0001	-0.0000	0.0027	-0.0003	0.0006
2B	50	0.0204	0.0326	0.0431	0.0566	0.0651	0.0638
2C	50	0.0304	0.0533	0.0742	0.0989	0.1473	0.1881
3	50	0.0032	0.0047	-0.0119	-0.0202	0.0968	0.0675
4	50	0.0015	-0.0003	-0.0001	0.0036	-0.0012	0.0008
1	100	-0.0004	0.0009	0.0003	-0.0017	0.0009	-0.0002
2A	100	-0.0005	0.0012	-0.0001	-0.0015	-0.0002	-0.0000
2B	100	0.0188	0.0341	0.0430	0.0498	0.0663	0.0632
2C	100	0.0290	0.0547	0.0742	0.0914	0.1493	0.1876
3	100	-0.0008	0.0018	-0.0091	-0.0103	0.0576	-0.0958
4	100	-0.0005	0.0014	-0.0002	-0.0016	-0.0007	0.0003
1	200	0.0008	0.0001	-0.0003	0.0006	0.0001	0.0001
2A	200	0.0005	0.0003	-0.0004	0.0007	0.0008	-0.0001
2B	200	0.0199	0.0336	0.0420	0.0515	0.0693	0.0627
2C	200	0.0301	0.0545	0.0725	0.0925	0.1522	0.1873
3	200	0.0009	-0.0002	-0.0100	0.0198	0.0082	-0.2721
4	200	0.0004	0.0004	-0.0005	0.0008	0.0016	-0.0003

Table C2: Average ratio $\frac{\hat{\sigma}}{\sigma}$ – double-sided truncation, Methods 1 (N no), 2A (N l_L/l_U), 2B (N $0.5l_L/1.5l_U$), 2C (N $\sqrt{0.5}l_L/(1+\sqrt{0.5})l_U$), 3 (TN), 4 (CN)

method	n	$p = 0.05$	$p = 0.1$	$p = 0.15$	$p = 0.2$	$p = 0.4$	$p = 0.6$
1	10	0.8099	0.7208	0.6577	0.6055	0.4020	0.2449
2A	10	0.8913	0.8443	0.8104	0.7737	0.6008	0.4227
2B	10	1.0201	1.0854	1.1462	1.1900	1.2837	1.2992
2C	10	1.0702	1.1686	1.2513	1.3092	1.4231	1.4147
3	10	1.4101	1.6947	1.9568	2.1934	2.2537	1.9379
4	10	0.9455	0.9497	0.9657	0.9798	1.0131	1.0159
1	50	0.8579	0.7746	0.7086	0.6533	0.4516	0.2871
2A	50	0.9404	0.8975	0.8560	0.8174	0.6328	0.4432
2B	50	1.1006	1.1822	1.2383	1.2918	1.3614	1.3787
2C	50	1.1735	1.2955	1.3740	1.4458	1.5174	1.5033
3	50	1.0131	1.0722	1.2927	1.5118	2.5724	2.4659
4	50	0.9855	0.9888	0.9917	1.0022	0.9979	1.0240
1	100	0.8641	0.7819	0.7166	0.6555	0.4578	0.2910
2A	100	0.9470	0.9033	0.8638	0.8190	0.6384	0.4449
2B	100	1.1158	1.1916	1.2525	1.2942	1.3776	1.3854
2C	100	1.1951	1.3085	1.3921	1.4479	1.5361	1.5109
3	100	1.0034	1.0168	1.0835	1.3406	3.0285	3.9334
4	100	0.9916	0.9918	0.9987	0.9985	1.0049	1.0120
1	200	0.8692	0.7868	0.7177	0.6602	0.4611	0.2951
2A	200	0.9532	0.9096	0.8623	0.8203	0.6400	0.4467
2B	200	1.1257	1.2047	1.2490	1.2924	1.3816	1.3907
2C	200	1.2074	1.3254	1.3891	1.4464	1.5430	1.5165
3	200	1.0055	1.0112	1.1008	2.1777	9.0896	9.7218
4	200	0.9982	0.9992	0.9925	0.9949	0.9994	1.0091

Table C3: MSE($\hat{\mu}$) – double-sided truncation, methods 1 (N no), 2A (N l_L/l_U), 2B (N $0.5l_L/1.5l_U$), 2C (N $\sqrt{0.5}l_L/(1+\sqrt{0.5})l_U$), 3 (TN), 4 (CN)

method	n	$p = 0.05$	$p = 0.1$	$p = 0.15$	$p = 0.2$	$p = 0.4$	$p = 0.6$
1	10	0.3248	0.2755	0.2530	0.2277	0.1519	0.0932
2A	10	0.3740	0.3414	0.2950	0.2748	0.1637	0.0749
2B	10	0.5247	0.6287	0.6581	0.7451	0.8927	0.8384
2C	10	0.6118	0.7983	0.8951	1.0502	1.5544	1.7664
3	10	67.2448	113.3622	173.3175	279.8236	661.8886	590.9577
4	10	0.4242	0.4349	0.4226	0.4678	0.5278	0.5358
1	50	0.0645	0.0538	0.0474	0.0439	0.0308	0.0192
2A	50	0.0703	0.0665	0.0602	0.0502	0.0354	0.0148
2B	50	0.1067	0.1424	0.1782	0.2056	0.2673	0.2416
2C	50	0.1338	0.2104	0.3017	0.4041	0.7412	1.0514
3	50	0.1432	1.7270	10.5357	18.3410	193.2746	282.9373
4	50	0.0775	0.0821	0.0833	0.0780	0.0979	0.0931
1	100	0.0311	0.0287	0.0246	0.0215	0.0135	0.0096
2A	100	0.0365	0.0339	0.0298	0.0267	0.0165	0.0082
2B	100	0.0606	0.0906	0.1113	0.1277	0.1889	0.1789
2C	100	0.0809	0.1505	0.2190	0.2912	0.6571	0.9754
3	100	0.0572	0.1017	1.4560	7.1483	138.4885	346.6464
4	100	0.0403	0.0415	0.0411	0.0405	0.0452	0.0507
1	200	0.0156	0.0148	0.0131	0.0109	0.0071	0.0040
2A	200	0.0179	0.0178	0.0162	0.0139	0.0084	0.0038
2B	200	0.0348	0.0589	0.0778	0.1011	0.1593	0.1350
2C	200	0.0515	0.1111	0.1729	0.2577	0.6280	0.9202
3	200	0.0282	0.0415	1.3600	36.0369	622.7075	1474.9191
4	200	0.0197	0.0217	0.0221	0.0210	0.0225	0.0231

Table C4: MSE($\hat{\sigma}$) – double-sided truncation, methods 1 (N no), 2A (N l_L/l_U), 2B (N $0.5l_L/1.5l_U$), 2C (N $\sqrt{0.5}l_L/(1+\sqrt{0.5})l_U$), 3 (TN), 4 (CN)

method	n	$p = 0.05$	$p = 0.1$	$p = 0.15$	$p = 0.2$	$p = 0.4$	$p = 0.6$
1	10	0.2711	0.4090	0.5504	0.6912	1.4783	2.3147
2A	10	0.1964	0.2127	0.2338	0.2719	0.6666	1.3424
2B	10	0.4745	0.5581	0.6197	0.6168	0.6116	0.5279
2C	10	0.7734	1.0022	1.1658	1.2103	1.2009	0.9238
3	10	14.9749	27.2921	37.2472	44.2286	43.8059	30.4259
4	10	0.2662	0.3029	0.3231	0.3792	0.6574	0.7669
1	50	0.1027	0.2192	0.3541	0.4918	1.2106	2.0371
2A	50	0.0405	0.0617	0.0978	0.1453	0.5435	1.2415
2B	50	0.1528	0.2470	0.3308	0.4359	0.5740	0.5982
2C	50	0.3196	0.5513	0.7436	0.9561	1.1496	1.0444
3	50	0.1667	1.6028	8.4019	13.1025	54.1006	37.6171
4	50	0.0449	0.0491	0.0508	0.0622	0.0926	0.1852
1	100	0.0847	0.1985	0.3282	0.4809	1.1790	2.0130
2A	100	0.0242	0.0476	0.0816	0.1369	0.5252	1.2332
2B	100	0.1125	0.2112	0.3076	0.3908	0.5972	0.6073
2C	100	0.2568	0.4962	0.7074	0.8786	1.1905	1.0611
3	100	0.0617	0.1498	1.2345	10.0949	71.8792	100.0286
4	100	0.0220	0.0251	0.0257	0.0273	0.0482	0.0861
1	200	0.0740	0.1859	0.3221	0.4645	1.1634	1.9882
2A	200	0.0153	0.0379	0.0797	0.1321	0.5194	1.2248
2B	200	0.0933	0.1976	0.2744	0.3655	0.5937	0.6168
2C	200	0.2260	0.4756	0.6520	0.8367	1.1968	1.0747
3	200	0.0304	0.0577	5.3261	67.2633	487.8735	500.9458
4	200	0.0109	0.0128	0.0132	0.0145	0.0192	0.0402

Appendix D Comparison of methods

Table D1: Comparison of methods based on different criteria, methods 1 (N no), 2A (N l_L/l_U), 2B (N $0.5l_L/1.5l_U$), 2C (N $\sqrt{0.5}l_L/(1+\sqrt{0.5})l_U$), 3 (TN), 4 (CN)

truncation	performance	$(\hat{\mu}-\mu)/\mu$	$\hat{\sigma}/\sigma$	MSE($\hat{\mu}$)	MSE($\hat{\sigma}$)
left-sided	best	4	4	2C	2B/2C
		2C	2B/2C	4	2B/2C
		2B	2B/2C	2B	4
		2A	2A	2A	1
		1	1	1	2A
	worst	3	3	3	3
right-sided	best	4	4	4	4
		2B	2A	2A	2A
		2A	1	2B	2B
		2C	2B	2C	1
		1	2C	1	2C
	worst	3	3	3	3
double-sided	best	1/2A/4	4	1/2A	4
(symmetric)		1/2A/4	2A	1/2A	2A
		1/2A/4	2B	4	2B
		2B	1	2B	1
		2C	2C	2C	2C
	worst	3	3	3	3
